# Prevention of quality decline and delivery of siRNA using exogenous TCTP translocation across the zona pellucida in mouse oocytes

**DOI:** 10.1038/s41598-019-55449-4

**Published:** 2019-12-11

**Authors:** Hyuk-Joon Jeon, Guang-Yu Bai, Yuram Park, Jae-Sung Kim, Jeong Su Oh

**Affiliations:** 1 Department of Integrative Biotechnology, College of Biotechnology and Bioengineering, Sungkyunkwan University, Suwon, Korea; 2 Biomedical Institute for Convergence at SKKU (BICS), Sungkyunkwan University, Suwon, Korea; 3 Division of Radiation Biomedical Research, Korea Institute of Radiological and Medical Sciences, Seoul, Korea

**Keywords:** Genetic transduction, Oogenesis

## Abstract

The delivery of exogenous molecules into mammalian oocytes or embryos has been a challenge because of the existence of the protective zona pellucida (ZP) surrounding the oocyte membrane. Here we show that exogenous translationally controlled tumor protein (TCTP) is able to translocate into oocytes across the ZP and prevents quality deterioration during *in vitro* culture. Recombinant TCTP-mCherry added to culture media were incorporated into oocytes after passing through the ZP. After internalization, recombinant TCTP-mCherry were enriched at the cortex with wide distribution within the cytoplasm. This translocation capacity of TCTP is dependent on its N-terminal protein transduction domain (PTD). Moreover, translocated recombinant TCTP-mCherry reduced quality deterioration of oocytes during prolonged *in vitro* culture, which in turn improved fertilization and early embryo development. Furthermore, conjugates between PTD of TCTP and cyclin B1 siRNAs internalized into the cytoplasm of oocytes and downregulated cyclin B1 level. Therefore, our results are the first to show that TCTP has the ability to translocate into oocyte cytoplasm penetrating through the ZP, providing the possibility for preserving oocyte quality during extended *in vitro* culture and for delivering siRNAs into mouse oocytes.

## Introduction

Mammalian oocytes are arrested at the metaphase of the second meiosis (MII) until fertilization. If fertilization does not occur for an extended time, oocytes experience time-dependent deterioration in quality^1,2^. In humans, a decline in oocyte quality is one of the major problems associated with failure in assisted reproductive technology (ART)^3^. Therefore, preserving oocyte quality during *in vitro* culture is important not only for ART success, but also for healthy reproduction.

We previously found that translationally controlled tumor protein (TCTP) has properties to relieve deterioration in oocyte quality and delay the onset of apoptosis during *in vitro* culture^4^. This suggested that TCTP could be a promising approach to decrease quality decline in oocytes cultured for a prolonged time during ART procedures. However, an invasive microinjection is unavoidable for clinical application of TCTP to penetrate the zona pellucida (ZP), a glycoprotein matrix that surrounds the plasma membranes of oocytes and embryos and that functions as a barrier to exogenous materials^5,6^. Recently, TCTP was found to contain a protein transduction domain (PTD) at its N-terminal^7,8^. Although many PTDs have been discovered and employed for delivery of cargo molecules into a variety of cell types, PTDs that penetrate the ZP of mammalian oocytes and embryos have not been well described. Therefore, in this study, we tested whether TCTP can penetrate the ZP of oocytes and prevent deterioration of quality during *in vitro* culture. We found that exogenous TCTP is translocated into the cytoplasm of oocytes across the ZP during *in vitro* culture and then prevents time-dependent decline in oocyte quality, which in turn improves fertilization capacity and subsequent embryo development. Moreover, conjugates between PTD of TCTP and siRNAs internalized into oocytes and downregulated the expression of the target protein. Therefore, our results demonstrate that non-invasive delivery of recombinant TCTP could be used as an anti-aging factor to prevent deterioration in oocyte quality during *in vitro* culture and a novel delivery system for siRNAs into oocytes.

## Results

### TCTP is internalized into the cytoplasm of oocytes across the zona pellucida

**Figure 1:**
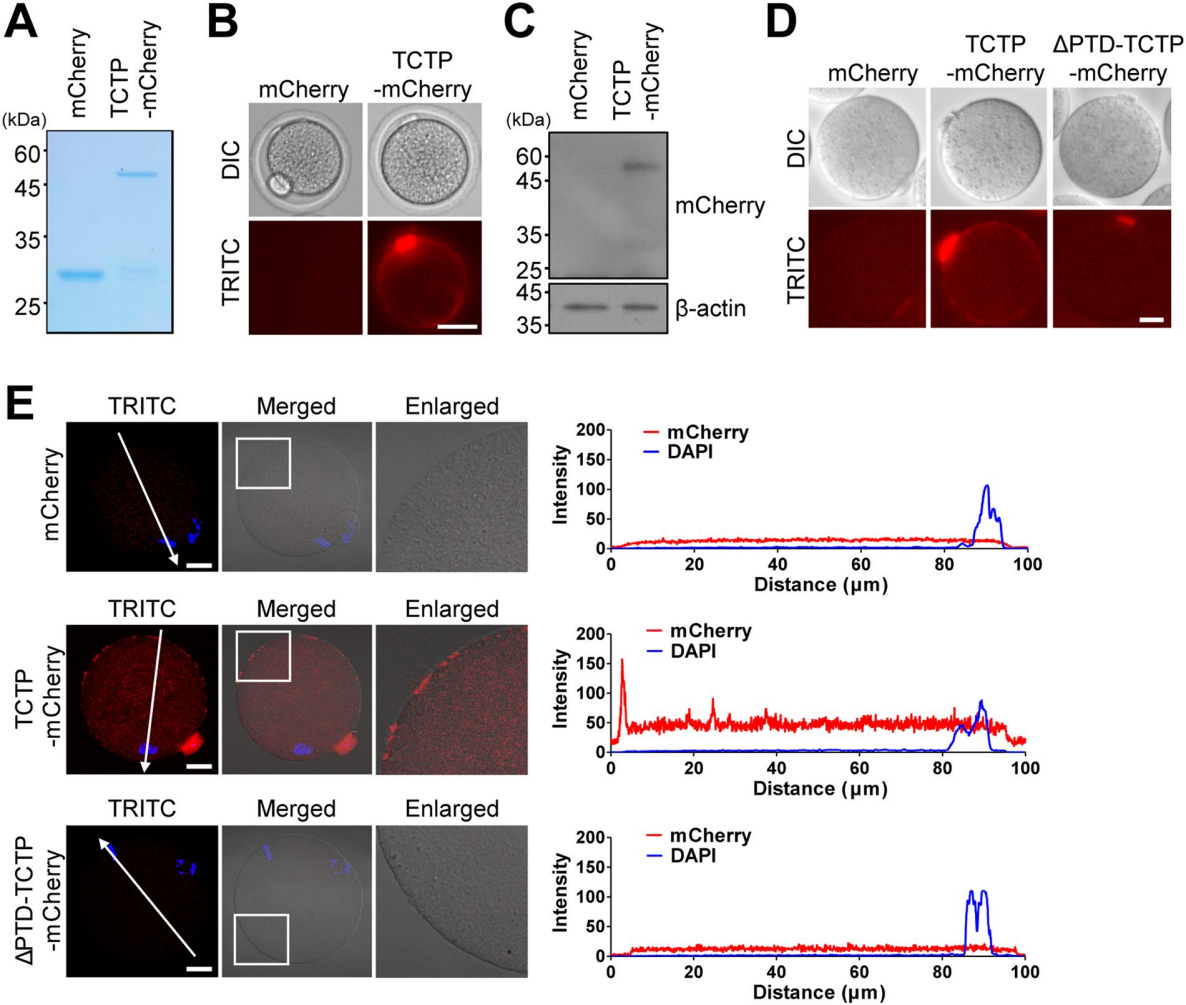
TCTP is internalized into the cytoplasm of oocytes across the zona pellucida (ZP). (**A**) SDS-PAGE of purified mCherry and TCTP-mCherry proteins with Coomassie staining. (**B**) Differential interference contrast (DIC) and TRITC fluorescence images of oocytes treated with 0.5 μM of mCherry or TCTP-mCherry proteins for 4 hours. Bar, 40 μm. (**C**–**E**) After treating oocytes with mCherry or TCTP-mCherry for 4 hours, the ZP was removed. (**C**) ZP-free oocytes were subjected to immunoblotting with mCherry antibody. Each lane contained 50 oocytes, and β-actin was used as a loading control. Cropped blots are represented and full-length blots are reported in Supplementary Fig. 1. (**D**) Representative live images of ZP-free oocytes pre-treated with mCherry or TCTP-mCherry. Bar, 20 μm. (**E**) Representative immunofluorescence images of ZP-free oocytes pre-treated with mCherry or TCTP-mCherry. Bar, 20 μm. Lines were drawn through the oocytes, and pixel intensities were quantified along the lines.

To investigate whether TCTP can penetrate the zona pellucida (ZP) of mouse oocytes, we purified recombinant TCTP proteins tagged with mCherry at the C-terminal (Fig. 1A). After treating with purified TCTP-mCherry proteins for 4 hours, oocytes at the MII stage were examined by fluorescence microscopy for direct visualization. Whereas no fluorescence signal was detectable in control oocytes treated with mCherry proteins, a strong signal was observed in the surroundings of oocytes treated with TCTP-mCherry proteins (Fig. 1B).

Because the TCTP-mCherry signal was localized to oocyte surfaces with enrichment at the polar body, it was of interest to determine whether TCTP was internalized into the cytoplasm of oocytes or if it simply coated oocyte surfaces after penetrating the ZP. To investigate this, the ZP was removed from oocytes at the MII stage after TCTP-mCherry treatment, and ZP-free oocytes were subjected to immunoblotting analysis. While no detectable signal was observed in oocytes treated with mCherry proteins, a clear mCherry signal was detected in oocytes treated with TCTP-mCherry proteins (Fig. 1C). To further clarify the incorporation of TCTP into oocyte cytoplasm, ZP-free MII oocytes treated with TCTP-mCherry were visualized under a fluorescence microscope. We found that the fluorescence signal remained detectable at the surface of oocytes treated with TCTP-mCherry proteins, while no signal was detected in control oocytes treated with mCherry proteins (Fig. 1D). Because TCTP contains the protein transduction domain (PTD) at its N-terminal, we sought to determine whether translocation of TCTP into mouse oocytes is mediated by its N-terminal PTD sequence. Interestingly, the fluorescence signal was barely detectable when oocytes at the MII stage were treated with TCTP-mCherry lacking PTD (∆PTD-TCTP-mCherry) (Fig. 1D). This result indicates that uptake of TCTP into oocytes is PTD-dependent. Confocal microscopic analyses further revealed that TCTP was internalized into the cytoplasm of oocytes with enrichment at the cortex (Fig. 1E). Therefore, our results demonstrate that TCTP-mCherry is internalized into the cytoplasm of oocytes after passing through the ZP.

### Exogenous TCTP relieves the decline of oocyte quality

**Figure 2:**
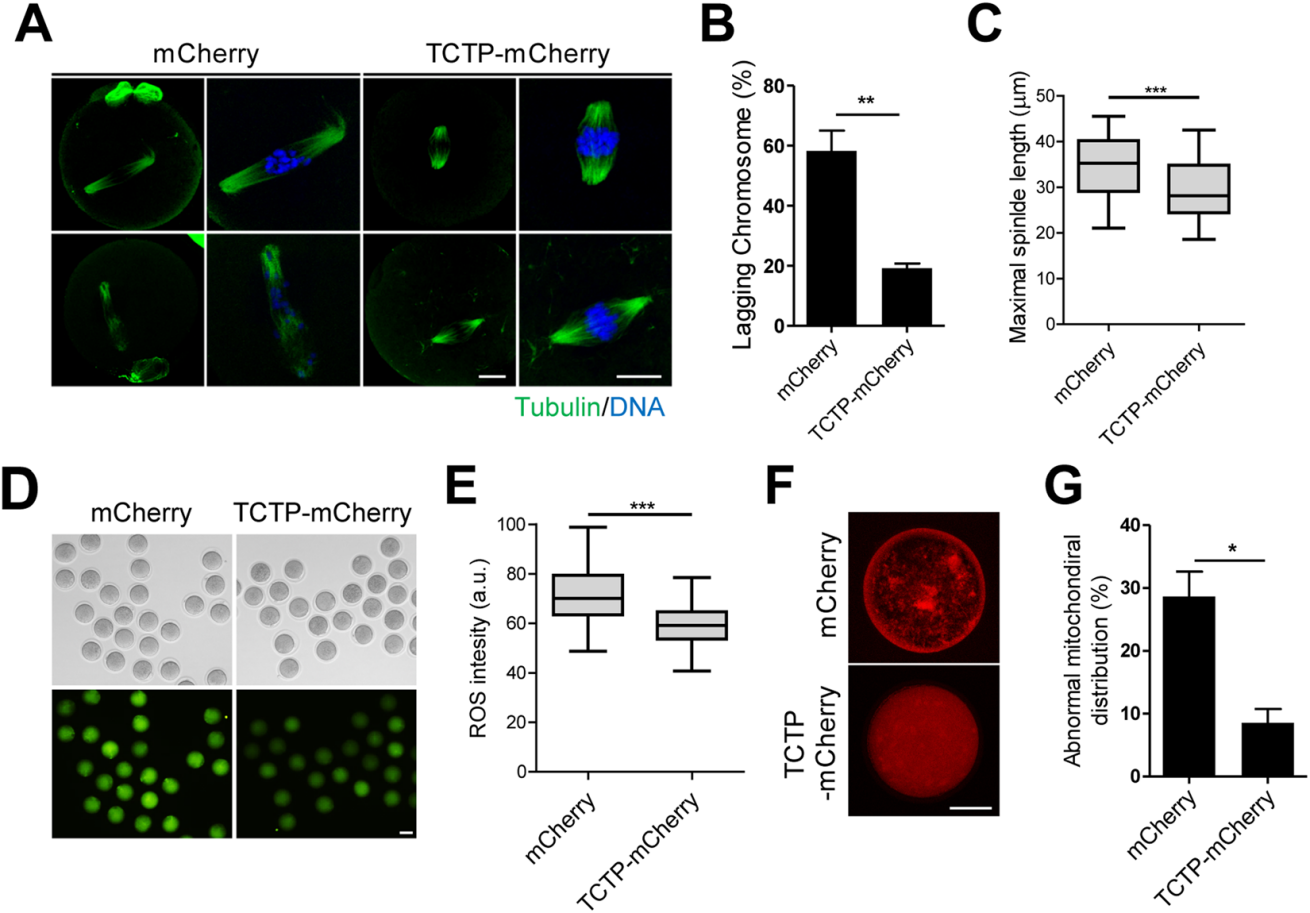
Exogenous TCTP relieves the decline of oocyte quality. Oocytes were treated with mCherry or TCTP-mCherry for 4 hours. After washing the proteins, oocytes were *in vitro* cultured in fresh media for 24 hours. (**A**–**C**) Spindle microtubules and chromosomes were immunostained with anti-tubulin antibody and DAPI, respectively. Representative images from three independent experiments are shown in (**A**). Bar, 20 μm. (**B**,**C**) Percentage of lagging chromosomes and maximal spindle length are shown. Data are expressed as means ± SEMs from three independent experiments. (**D**,**E**) Oxidative stress levels were determined by measuring fluorescence intensity following incubation with DCF-DA for 30 min. Representative images from three independent experiments are shown, along with quantification of ROS levels. Bar, 80 μm. (**F**,**G**) Mitochondrial distribution was determined by immunostaining with CytoPainter MitoRed. Representative images from three independent experiments are shown. Bar, 40 μm. The incidence of mitochondrial aggregation is shown. Data are expressed as means ± SEMs from three independent experiments. *p < 0.05, **p < 0.001, ***p < 0.0001 (Student’s t-test).

We previously reported that overexpression of TCTP prevented the decline of oocyte quality during prolonged *in vitro* culture^4^. Thus, we examined whether exogenous TCTP-mCherry proteins are capable of improving oocyte quality after internalizing into the cytoplasm of oocytes. To this end, we cultured mouse MII oocytes for 24 hours after pre-treating them with TCTP-mCherry proteins. Confocal microscopy analysis revealed that the incidence of lagging chromosomes was dramatically increased in control oocytes after 24 hours of culture. However, pre-treatment with TCTP-mCherry reduced the incidence of chromosome lagging (2A, B). Moreover, the spindles became elongated or disorganized in control oocytes after 24 hours of culture, but these abnormalities in spindle organization decreased by pre-treating oocytes with TCTP-mCherry proteins (Fig. 2A,C). To further investigate the effects of exogenous TCTP-mCherry protein treatment, we examined oxidative stress and mitochondrial aggregation, which are the main causes of quality deterioration in oocytes^9^. While reactive oxygen species (ROS) levels and mitochondria aggregation increased after 24 hours of *in vitro* culture in control oocytes treated with mCherry proteins, oocytes treated with TCTP-mCherry proteins showed significant reduction in ROS levels and mitochondria aggregation (Fig. 2D–G). Therefore, our results demonstrate that exogenous TCTP-mCherry proteins have the capacity to relieve the deterioration of oocyte quality during *in vitro* culture after internalization.

### TCTP treatment during *in vitro* aging enhances fertilization and early embryo development

**Figure 3:**
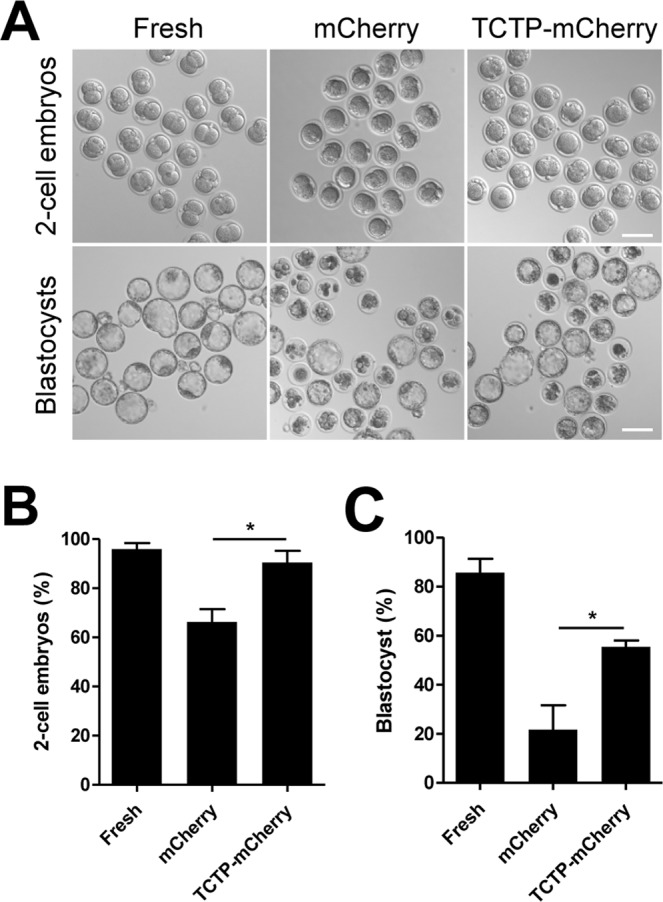
TCTP treatment enhances fertilization and early embryo development. TCTP treatment improves preimplantation embryo development. Oocytes pre-treated with mCherry or TCTP-mCherry were inseminated by ICSI after 12 hours of *in vitro* culture. As a control, fresh oocytes were inseminated by ICSI. (**A**) Representative images of 2-cell embryos and blastocysts are shown. Bar, 100 μm. (**B**,**C**) The rate of 2-cell and blastocyst development is shown. *p* < *0.05 (one-way ANOVA followed by Newman-Keuls post-hoc test).

To expand upon our findings, we further investigated whether oocyte quality improvement by TCTP treatment during *in vitro* culture enhances fertilization and subsequent embryo development. Oocytes at the MII stage were cultured *in vitro* for 12 hours with either mCherry or TCTP-mCherry proteins and then fertilized using intracytoplasmic sperm injection (ICSI). Fresh MII oocytes were used as a control. The rate of 2-cell and blastocyst development was significantly reduced in oocytes treated with mCherry proteins during *in vitro* culture compared to fresh oocytes, which is consistent with quality degeneration during *in vitro* aging^1,2^. However, the rate of 2-cell and blastocyst development significantly improved after treatment with TCTP-mCherry proteins (Fig. 3A–C). Therefore, our data show that exogenous TCTP effectively prevents the decline in egg quality during *in vitro* aging and thereby improves subsequent fertilization and early embryo development.

### PTD of TCTP efficiently delivers siRNAs into mouse oocytes

**Figure 4:**
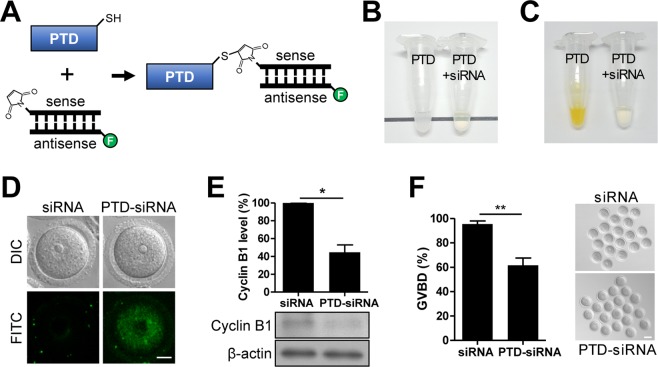
Conjugates between PTD of TCTP and siRNAs are effectively delivered into mouse oocytes. (**A**) Synthesis of conjugates between PTD of TCTP and siRNAs via a thiol-maleimide coupling. (**B**) After conjugation between PTD and siRNA, the transparency of reaction mixtures was shown. (**C**) The conjugation between PTD and siRNA was determined by Ellman’s test. (**D**) The uptake of PTD-siRNA conjugates was determined by confocal microscopy after treating GV oocytes with PTD-siRNA for 4 hours. Bar, 20 μm. (**E**) GV oocytes were treated with either unconjugated cyclin B1 siRNAs or PTD-cyclin B1 siRNAs for 24 h with IBMX. Knockdown of cyclin B1 was confirmed by immunoblotting analysis. β-actin was used as a loading control. Each lane contains 40 oocytes. Quantification of cyclin B1 levels was shown. Cropped blots are represented and full-length blots are reported in Supplementary Fig. 2. (**F**) After 24 hours treatment with either unconjugated cyclin B1 siRNAs or PTD-cyclin B1 siRNAs, oocytes were released from IBMX-mediated GV arrest and GV breakdown (GVBD) was scored after 4 hours. Representative images from three independent experiments are shown, along with quantification of GVBD rate. Bar, 80 μm. The data are expressed as mean ± SEM from three independent experiments. *p < 0.05, **p < 0.001 (Student’s t-test).

Delivery of siRNAs into mammalian oocytes or embryos has been a challenge because of the existence of the protective ZP surrounding the oocyte membrane. Because TCTP penetrated the ZP and internalized into the cytoplasm in mouse oocytes, we tested whether TCTP could be used to deliver siRNAs into mouse oocytes. To this end, fluorescence dye-labeled siRNAs targeting cyclin B1 were conjugated to the PTD of TCTP (PTD-siRNA) (Fig. 4A). Successful conjugation between PTD and siRNAs was determined by increased solubility and Ellman’s test. While the PTD itself was insoluble in PBS due to its hydrophobic property, the siRNA-conjugated PTD was solubilized in PBS (Fig. 4B). Moreover, the disappearance of thiol group in PTD by Ellman’s test showed that cyclin B1 siRNA and PTD were successfully conjugated (Fig. 4C). We next determined the uptake of siRNAs after treating GV oocytes with PTD-siRNA conjugates for 4 hours. Whereas no fluorescence signal was detected in control oocytes treated with unconjugated siRNAs, the fluorescence signal was clearly observed in oocytes treated with PTD-siRNA conjugates (Fig. 4D). This result suggests that siRNAs were successfully internalized into the cytoplasm of mouse oocytes.

To further investigate whether the uptake of siRNAs mediated by conjugation with PTD of TCTP is sufficient to knockdown target gene expression in mouse oocytes, we cultured GV oocytes with PTD-siRNA conjugates for 24 hours and determined cyclin B1 level and GVBD rate. Immunoblot analysis showed that cyclin B1 level was decreased after PTD-siRNA conjugate treatment (Fig. 4E). Consistent with this, the rate of GVBD was decreased in oocytes treated with PTD-siRNA conjugates (Fig. 4F). Taken together, our results demonstrate that PTD-siRNA conjugates can be effectively delivered into oocytes and successfully target specific gene expression.

## Discussion

In this study, we found that TCTP has the ability to translocate into the cytoplasm of mouse oocytes across the ZP and to prevent deterioration in quality during *in vitro* culture. Moreover, we demonstrated that the conjugation between PTD of TCTP and siRNAs could be used for non-invasive siRNA delivery system into mouse oocytes.

The ZP is an extracellular glycoprotein matrix that surrounds all mammalian oocytes and plays a critical role in fertilization^6^. Although a number of proteins containing PTD have been identified, PTDs that can penetrate the ZP of mammalian oocytes have not been well studied. In this study, we observed for the first time that exogenous TCTP proteins translocated across the ZP and was internalized into oocyte cytoplasm. Once internalized, TCTP was enriched at oocyte cortex with wide distribution in the cytoplasm. This may be partly due to the presence of cortical granules, which are densely localized along the oocyte periphery^10^. We found that TCTP-mCherry proteins lacking PTD were not able to translocate into oocytes, suggesting that the translocation capacity of TCTP into mouse oocytes is dependent on its N-terminal sequence. Consistent with our findings, a hydrophobic peptide, MIIYRDLISH, located at the N-terminus of TCTP is known to contain PTD activity in a variety of cell types^7,8^. However, the molecular mechanisms of TCTP translocation are unclear. Given that TCTP is internalized via lipid-raft/caveola-dependent endocytosis and micropinocytosis in a dynamin/actin/microtubule-dependent pathway in human lung carcinoma cells^11^, it is likely that TCTP is internalized into the cytoplasm of oocytes via endocytosis. However, we could not exclude the possibility that TCTP translocation into oocytes is endocytosis-independent, because certain proteins containing PTD utilize non-endocytic mechanisms for cellular internalization^12,13^. On the other hand, the mechanism to penetrate the ZP seems to be different from that of translocation across the membranes. Given that the ZP is an extracellular glycoprotein matrix, it is tempting to speculate that TCTP interacts with carbohydrate residues of the ZP in mouse oocytes. Consistent with this, some PTDs have been shown to interact electrostatically with the extracellular matrix of the cells, followed by endocytosis^14^. Similarly, internalization of Tat protein is dependent on an interaction with cell surface heparan sulfate proteoglycans^15^. Thus, future studies are required to verify the molecular mechanisms of TCTP internalization into oocytes across the ZP.

There is a growing demand for assisted reproductive technologies (ART) in response to increasing infertility rates. However, reduced oocyte quality during extended culture for an ART procedure is a major obstacle to success. We previously showed that TCTP has properties to relieve deterioration in oocyte quality and delay the onset of apoptosis during *in vitro* culture. Therefore, we investigated whether it is possible to prevent oocyte quality degeneration during *in vitro* culture by adding exogenous TCTP to the culture media. Our results showed that treating oocytes with exogenous TCTP was sufficient to attenuate quality decline during prolonged *in vitro* culture. This suggests that TCTP maintains physiological activity after translocation into oocytes. Our results provide a potential avenue for improving oocyte quality during ART procedures. However, it should be noted that ZP glycoproteins are species-specific. For example, the human ZP contains four glycoproteins, ZP1 to ZP4, instead of the three ZP proteins found in mouse oocytes^5^. Therefore, it is important to determine whether TCTP can penetrate other mammalian ZPs, including human oocytes.

In conclusion, our results demonstrate for the first time that TCTP has the ability to translocate into the cytoplasm of mouse oocytes across the ZP and to preserve oocyte quality during extended *in vitro* culture. Given that the delivery of large molecules into mammalian oocytes and embryos is largely dependent on an invasive microinjection technique, our results provide the possibility that conjugates between PTD of TCTP and other molecules, including nucleic acids and proteins, will be applicable for new delivery systems for mammalian oocytes and embryos. This will improve not only ART procedures, but also transgenic technology by presenting a new paradigm to establish a non-invasive approach for delivering various molecules into mammalian oocytes and embryos.

## Materials and Methods

### Plasmid construction and protein purification

Plasmid pRN3-TCTP-mCherry was described previously^16^. The coding sequences of mCherry, TCTP-mCherry, and ΔPTD-TCTP-mCherry were amplified by PCR using the pRN3-TCTP-mCherry vector as a template and were cloned into the pET-26b (+) vector. The primers used for amplification are listed. mCherry, GAGATATACATATGATTATCTACCGGGAC and TGGTGGTGCTCGAGAGTCTTGTACAGCTCG; TCTP-mCherry, GAGATATACATATGGTGAGCAAGGGCG and TGGTGGTGCTCGAGAGTCTTGTACAGCTCG; ΔPTD-TCTP-mCherry, GAGATATACATATGTTCTCCGACATCTAC and TGGTGGTGCTCGAGTGGACATTTTTCCATTTC.

The expression plasmids were transformed into *E*. *coli* BL21(DE3). Expression and purification of recombinant proteins were carried out using Ni-NTA agarose resins (Thermo Fisher Scientific) according to the manufacturer’s protocol. Briefly, exponentially growing LB cultures (OD_600_ = 0.6) were induced using 0.5 mM isopropyl thio-β-D-galactopyranoside (IPTG) at 25 °C. After 24 hours, cultures were harvested and lysed by sonication. After centrifuging the lysates, the cleared supernatant was incubated and gently rotated overnight at 4 °C with Ni-NTA agarose resins. After washing with a washing buffer (0.05 M Tris-HCl, 0.3 M NaCl, 10 mM imidazole, pH 8.0), the proteins were eluted from the Ni-NTA agarose resins with an elution buffer (0.05 M Tris-HCl, 0.3 M NaCl, 250 mM imidazole, pH 8.0). The purified proteins in the elution buffer were dialyzed in PBS and concentrated using centrifugal filter units with an ultracel-10 membrane (Merck Millipore).

### Oocyte collection and culture

All procedures regarding mouse care and use were conducted in accordance with guidelines and approved by the Institutional Animal Care and Use Committees of Sungkyunkwan University (approval ID: SKKUIACUC2018-04-01-3). Six-week-old CD-1 female mice (Koatech, Korea) were superovulated by injection of 5 IU of pregnant mare serum gonadotropin (PMSG; Sigma) followed by 5 IU of human chorionic gonadotropin (hCG; Sigma) 46–48 hours later. To obtain MII stage oocytes, oocyte-cumulus complexes were collected from the oviducts after another 13 hours, and cumulus cells were removed using hyaluronidase (Sigma). For germinal vesicle (GV) stage oocytes, immature oocytes were collected from the follicles 46–48 hours after PMSG injection and recovered in M2 medium (Zenith Biotech) supplemented with 100 μM 3-isobutyl-1-methylxanthine (IBMX) to prevent GV breakdown (GVBD). Cumulus-free oocytes were cultured in fresh M2 media in a 5% CO_2_ atmosphere at 37 °C. For protein treatment, purified recombinant proteins (0.5 μM) were added to the culture media and oocytes were cultured for 4 hours with recombinant proteins. In some experiments, the ZP was digested with acidic Tyrode’s solution (Sigma) before microscopic analysis.

### Immunoblotting analysis

Lysates from 40 oocytes in SDS sample buffer were subjected to SDS-PAGE and transferred to membrane. The membranes were incubated in the blocking buffer (0.1% Tween-20, 3% BSA in TBST) at room temperature for 1 hour and then with primary antibodies overnight at 4 °C. After extensive washing in TBST, membranes were incubated with HRP-labeled secondary antibodies (Jackson ImmunoResearch) for 2 hours. The membranes were developed with the ECL Plus Western Blotting Detection kit (GE Healthcare Life Science). Signals were quantified densitometrically using ImageJ software (National Institutes of Health) and expressed as relative values (i.e. normalized to the corresponding β-actin signal of the same membrane). The primary antibodies used for immunoblotting were anti-mCherry (1:500; Abcam), anti-cyclin B1 (1:1000; Cell Signaling), and anti-β-actin (1:2500; Cell Signaling).

### Immunofluorescence staining

Immunofluorescence staining was performed as described previously^4^. Briefly, oocytes were fixed in freshly prepared 4% paraformaldehyde in PBS for 20 min, washed in PBS, and then permeabilized with 0.1% Triton X-100 in PBS for 30 min. After incubating in blocking buffer (3% BSA in PBS) for 1 hour, immunostaining was performed using primary antibodies against acetylated α-tubulin (1:1000; Sigma), followed by Alex Fluor 488-conjugated secondary antibodies (Jackson ImmunoResearch). After counterstaining DNA with DAPI, oocytes were examined with an LSM 700 laser-scanning confocal microscopy (Zeiss) equipped with a C-Apochromat 40×/1.2 water immersion objective. Data were analyzed using ZEN 2010 LSM software (Zeiss) and ImageJ software (National Institutes of Health). For lagging chromosome analysis, oocytes with chromosomes aligned at the spindle equator were considered normal, while oocytes with either imperfectly aligned or clumped chromosomes were considered abnormal. All images were reviewed by two investigators in a double-blind manner.

### Mitochondria aggregation analysis

For visualizing mitochondria, oocytes were incubated with CytoPainter MitoRed (Abcam) for 30 min. After extensive washing, the oocytes were examined with an LSM 700 laser-scanning confocal microscopy. Data were analyzed using ImageJ software (National Institutes of Health).

### Measurement of intracellular reactive oxygen species (ROS) levels

Intracellular ROS levels were detected as described previously^4^. Briefly, oocytes were incubated with 10 μM of 2′,7′-dichlorofluorescin diacetate (DCF-DA; Sigma) for 30 min. After acquiring the fluorescence signals using a Nikon Eclipse Ti inverted microscope with a CCD-cooled camera (Nikon), the intensity of fluorescence was quantified using ImageJ software (National Institutes of Health).

### Synthesis of PTD-siRNA conjugates

Peptides corresponding to PTD of TCTP (MIIYRDLISH) with C-terminal modifications were commercially synthesized by Peptron Inc. (Deajeon, Korea) at a purity grade of >99%. Briefly, the peptides were synthesized using Fmoc solid phase peptide synthesis and purified using reverse phase high-performance liquid chromatography. After adding min-PEG2 chains, cysteine residue was introduced at the C-terminal of peptides for conjugation with siRNAs. Modified siRNAs targeting cyclin B1 were synthesized by Bioneer Co. (Deajeon, Korea). A thiol-reactive maleimide group was linked to the 5′ end of the sense strand of siRNA (5′-CCACCUGGAAAAGAAUCCU-3′) and fluorescent dye (FAM) was labeled at the 5′ end of the antisense strand of siRNA (5′-AGGAUUCUUUUCCAGGUGG-3′).

Conjugates between PTD peptides and cyclin B1 siRNAs were prepared using the thiol-maleimide reaction as described previously^17^. Briefly, PTD peptides (0.15 μM dissolved in DMSO) and siRNAs (0.5 μM dissolved in RNase-free distilled water) were incubated in 200 μl of PBS (pH 7.4) for 1 hour at 4 °C. After centrifugation, the clear supernatant was removed and stored frozen at −80 °C until use. The conjugation between PTD and siRNA was determined by Ellman’s test. For treatment, conjugates were diluted to 1:25 in M2 medium.

### Intracytoplasmic sperm injection (ICSI)

ICSI experiments were performed using spermatozoa obtained from the cauda epididymis of a BDF1 male as described previously^4,18^. Briefly, spermatozoa were placed in droplets of 10% polyvinyl pyrrolidone (PVP)-M2 media (Zenith Biotech) in a micromanipulation chamber. A single spermatozoa was sucked into an injection pipette and the head was separated from the tail by applying a few piezo pulses to the head-tail junction. The isolated sperm head was then injected into an oocyte retrieved from a BDF1 female mouse. The injected oocytes were kept in M2 media for 10 min at room temperature for recovery and cultured in KSOM media at 37 °C in a 5% CO_2_ atmosphere. In these experiments, aged oocytes were used only if they had normal surface features and were not spontaneously activated. After observing pronuclear formation, percentages of sperm-injected and surviving oocytes that reached the 2-cell and blastocyst stages were assessed after 22–24 and 96–98 hours, respectively.

### Statistical analysis

All statistical analysis was performed using GraphPad Prism 5.0 (GraphPad Software). Data are representative of at least three independent experiments unless otherwise specified, and at least 20 oocytes were examined for each group. Student’s t-test was used to assess differences between two groups, and one-way ANOVA with the Newman-Keuls post-hoc test was used to assess differences among three groups; p < 0.05 was considered statistically significant.

## Supplementary information

Supplementary Figures
