# Supplementary material for: Prevention of quality decline and delivery of siRNA using exogenous TCTP translocation across the zona pellucida in mouse oocytes
Source: Sci Rep. 2019 Dec 11;9:18845. doi: 10.1038/s41598-019-55449-4 (PMC6906282; doi:10.1038/s41598-019-55449-4)
Supplement: Supplementary file 1 — Supplementary Figures [file 41598_2019_55449_MOESM1_ESM.pdf]

Supplementary Information

Prevention of quality decline and delivery of siRNA using exogenous TCTP translocation across the zona pellucida in mouse oocytes

Hyuk-Joon Jeon[1], Guang-Yu Bai[1,2], Yuram Park[1], Jae-Sung Kim[3],*, and Jeong Su Oh[1,2,*]

[1]Department of Integrative Biotechnology, College of Biotechnology and Bioengineering, Sungkyunkwan University, Suwon, Korea

[2]Biomedical Institute for Convergence at SKKU (BICS), Sungkyunkwan University, Suwon, Korea

[3]Division of Radiation Biomedical Research, Korea Institute of Radiological and Medical Sciences, Seoul, KoreaTX

Corresponding authors: Department of Integrative Biotechnology, College of Biotechnology and Bioengineering, Sungkyunkwan University, Suwon, Korea. Email: ohjs@skku.edu; Division of Radiation Biomedical Research, Korea Institute of Radiological and Medical Sciences, Seoul, Korea. Email: jaesung@kirams.re.kr

Supplementary Figure 1

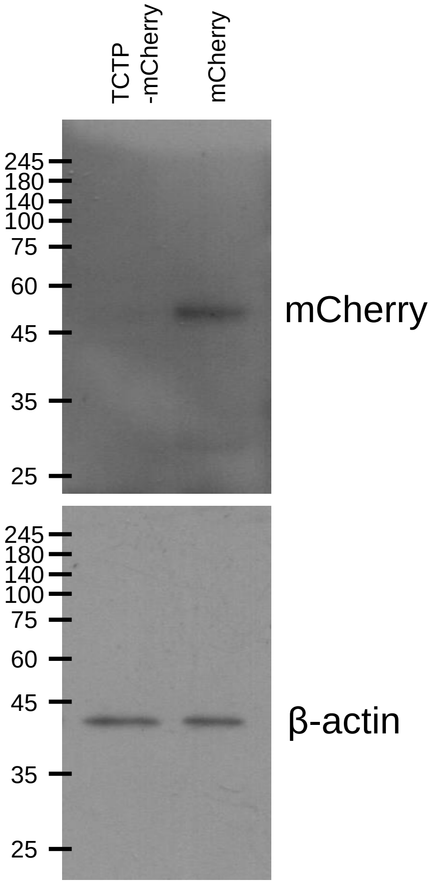

Supplementary Figure 1. Full-length blots for Fig. 1C

Supplementary Figure 2

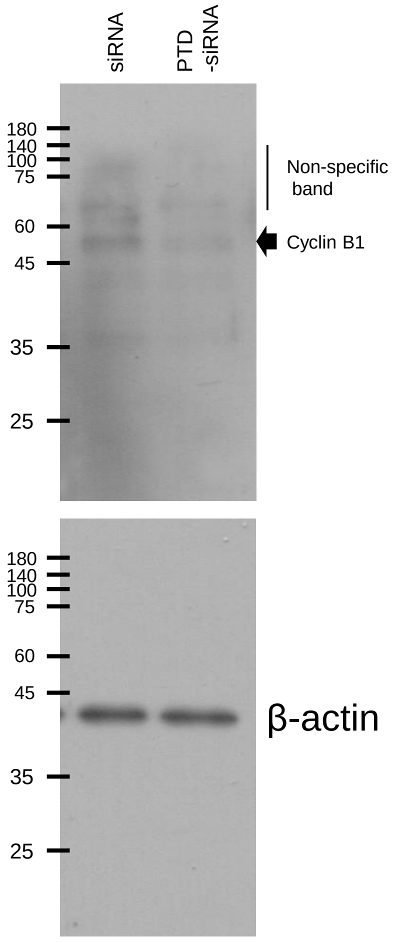

Supplementary Figure 2. Full-length blots for Fig. 4E.
